# Assessment of *Plasmodium falciparum *resistance to ferroquine (SSR97193) in field isolates and in W2 strain under pressure

**DOI:** 10.1186/1475-2875-5-11

**Published:** 2006-02-07

**Authors:** Wassim Daher, Christophe Biot, Thierry Fandeur, Helene Jouin, Lydie Pelinski, Eric Viscogliosi, Laurent Fraisse, Bruno Pradines, Jacques Brocard, Jamal Khalife, Daniel Dive

**Affiliations:** 1 Inserm U547, Institut Pasteur, 1 rue du Pr Calmette, B.P. 245, 59019 Lille, France; 2 Unité de Catalyse et Chimie du Solide – UMR CNRS 8181 Synthèse Organométallique et Catalyse, Ecole Nationale Supérieure de Chimie de Lille, 59652 Villeneuve d'Ascq cedex, France; 3 UMR Université-INRA d'Immunologie Parasitaire, Faculté des Sciences Pharmaceutiques, 31, avenue Monge, Parc Grandmont, 37200 Tours, France; 4 Immunologie Moléculaire des Parasites, Institut Pasteur, 25 rue du Dr Roux, 75724 Paris cedex 15, France; 5 Sanofi-Aventis Recherche, Discovery Department, 31000 Toulouse Cedex, France; 6 Institut de Médecine Tropicale du Service de Santé des Armées, Unité de Parasitologie, Bd Charles Livon, Parc le Pharo, BP 46, 13998 Marseille Armées, France

## Abstract

**Background:**

Ferroquine (FQ), or SSR97193, is a novel antimalarial drug currently in phase I clinical trials. FQ is a unique organometallic compound designed to overcome the chloroquine (CQ) resistance problem. FQ revealed to be equally active on CQ-sensitive and CQ-resistant *Plasmodium falciparum *laboratory strains and field isolates. FQ is also curative on rodent malaria parasites. As FQ will be tested in patients, the potential for resistance to this drug was evaluated.

**Methods:**

The relationship between CQ-resistant transporter gene genotype and susceptibility to FQ were studied in 33 Cambodian *P. falciparum *field isolates previously studied for their *in vitro *response to CQ. In parallel, the ability of the CQ-resistant strain W2, to become resistant to FQ under drug pressure was assessed.

**Results:**

The IC_50 _values for FQ in field isolates were found to be unrelated to mutations occurring in the *P. falciparum *chloroquine resistance transporter (PfCRT) or to the level of expression of the corresponding mRNA. *In vitro*, under a drug pressure of 100 nM of FQ, transient survival was observed in only one of two experiments.

**Conclusion:**

Field isolates studies and experimental drug pressure experiments showed that FQ overcomes CQ resistance, which reinforces the potential of this compound as a new antimalarial drug.

## Background

Drug resistance, particularly to CQ is an important limit to the control of *P. falciparum*, mainly in sub-Saharan Africa and South-east Asia. CQ is believed to act by concentrating in the parasite digestive vacuole and inhibiting the mechanism of detoxification of ferriprotoporphyrin IX that is produced during the digestion of haemoglobin, leading to parasite death. This detoxification takes place in the food vacuole and partly in the cytosol [1,2]. It was shown that CQ-resistant parasites expelled much more rapidly the CQ from RBC than CQ-sensitive parasites, and many observations indicated that a *P. falciparum *transmembrane protein (PfCRT) was involved in this efflux [3-7]. Mutations of PfCRT have been described in all CQ-resistant *P. falciparum *isolates. Moreover, the reduction of PfCRT expression *in vitro *by genetic manipulation in *P. falciparum *resulted in an increase in CQ susceptibility [8]. The genetic profile of CQ resistance in malaria parasites showed a particular mutation in PfCRT (K76T) that has been associated with CQ resistance in genetically modified *P. falciparum *strains and in field isolates [4-6].

**Figure 1:**
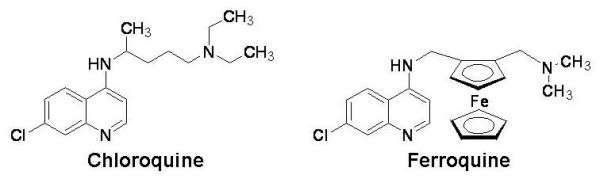
Structure of chloroquine and ferroquine.

Due to the ability of *Plasmodium *to develop resistance to antimalarial agents, an extensive search for new compounds has been initiated. An atypical strategy based on the incorporation of a metallocenic moiety into the CQ skeleton (Figure 1) has led to the identification of FQ (SSR97193), a new drug candidate exhibiting a powerful anti-malarial activity. Indeed, FQ was more potent than CQ in the inhibition of growth of *P. falciparum in vitro *and on *P. berghei in vivo *[9-11]. Recent studies, evaluating the *in vitro *susceptibility of African field isolates to FQ revealed that FQ IC_50 _ranged from 1 to 62 nM (8 to 1007 nM for CQ) in Franceville and Bakoumba (south-east Gabon) [14], between 0.43 to 30.9 nM in 103 isolates where 95% were resistant to CQin Libreville (Gabon) [15], and from 0.55 to 28.2 nM in 55 isolates where 55% of isolates were CQ-resistant in Senegal [13]. In these studies, a correlation was found between responses to FQ and CQ.

Incidently, a previous study performed on Cambodian isolates with different levels of *in vitro *resistance to CQ failed to reveal an association between the K76T mutation in PfCRT protein and susceptibility to FQ, although a correlation was observed between *in vitro *responses to both drugs [17]. Sequencing full length *pfcrt *cDNA of 42 isolates showed that other mutations than those already described can occur in the PfCRT protein. Six different PfCRT haplotypes were identified but the relation between these PfCRT haplotypes and susceptibility to FQ of isolates was not examined [18].

Ferroquine is currently in Phase 1 trials and its activity on *P. falciparum *uncomplicated malaria is planned to be evaluated in patients. For this reason, it was of interest to investigate whether a potential resistance to this drug may occur. For this purpose, a compilation was made of 155 results obtained from Cambodian isolates tested for their susceptibilities to CQ and FQ. Among them, *pfcrt *gene sequence and mRNA level expression were available for 33 isolates showing a large variation in FQ IC_50_. This led us, on one hand, to search a possible correlation between all mutations occurring in PfCRT protein and FQ susceptibility. On the another hand, we investigated wether the expression level of this gene may affect response to FQ. In parallel, the W2 strain, known to present a high capacity to acquire drug resistance, was submitted to FQ pressure at a sub-lethal dose [19-21].

## Methods

### Reagents

Chloroquine diphosphate was purchased from Sigma. Ferroquine (SSR97193) base was obtained from Sanofi Aventis (France) and RPMI 1640 culture medium was purchased from Invitrogen. Human erythrocytes and plasma were obtained through the EFS (Etablissement Français du Sang, France).

### Parasites

The 3D7, HB3 and Dd2 strains of *P. falciparum *used as a control for sensitivity to CQ and FQ were cultured as previously described [22]. For drug pressure experiments, a subclone of *P. falciparum *clone W2 was provided by Dr. B. Pradines (URBEP, Marseille, France). Parasites were grown *in vitro *in O^+ ^human red blood cells in RPMI 1640 medium (Invitrogen) supplemented with 10% AB human serum (EFS), and 0.01 mg/ml gentamicin under an atmosphere 90% nitrogen/5% oxygen/5% carbon dioxide.

### Field isolates

Complete data concerning CQ IC_50_, FQ IC_50 _and PfCRT protein sequences and *pfcrt *mRNA expression level were available for 33 of 155 patients previously recruited for *in vivo *trials of mefloquine-artesunate, by the Cambodian Ministry of Health. For these 33 patients a study concerning the relation between PfCRT mutations, expression of *pfcrt *mRNA and CQ resistance had already been performed [17,18].

### Antimalarial activity measurements

A microplate assay measuring [^3^H] hypoxanthine incorporation in parasite nucleic acids and derived from the method of Desjardins was used to test FQ antimalarial properties on laboratory strains [23]. *P. falciparum *laboratory strains, were selected on the basis of their susceptibility to CQ (3D7 and HB3 were sensitive, Dd2 was moderately resistant and W2 was resistant). Culture conditions and test procedures were as previously described (0.5% haematocrit; 0.5% parasitaemia) [11,12]. Ranges of drug concentrations used were 3.9–500 nM for CQ and 1.95–250 nM for FQ. IC_50 _were calculated from response curves by linear interpolation.

### Nucleic acid extraction from W2 clone and PfCRT polymorphism

W2 clone RNA was extracted from freshly infected erythrocytes with an Rneasy extraction kit (Quiagen). The *pfcrt *gene was amplified by reverse transcription (RT)-PCR and then sequenced, as previously described [24].

### Phylogenetic analysis of the PfCRT protein

In order to obtain a more accurate phylogeny of the PfCRT protein, a Bayesian phylogenetic analysis of PfCRT sequences was performed. For 3D7, Dd2 and HB3 clones, sequences were obtained from databanks (GenBank accession numbers: 3D7: CAD50842; Dd2: AAF26926; HB3: AAF60275). PfCRT sequences from field isolates were reported previously [18]. In total, 37 full-length PfCRT amino acid sequences were aligned using the BioEdit v7.0.1 package [25]. Alignment was facilitated by the fact that i) PfCRT protein sequences were extremely conserved (96–98% identity) and ii) none of these sequences exhibited any gaps or insertions, yielding 427 sites for analysis. The alignment used in this study is available upon request to the corresponding author. Phylogenetic analysis of this protein data set was carried out using software "MrBAYES", version 3_0b4 [26]. Bayesian analysis was performed using the Jones-Taylor-Thornton (JTT) amino acid replacement model [27]. Starting trees were random, four simultaneous Markov chains were run for 100,000 generations, burn-in values were set at 15,000 generations (based on empirical values of stabilizing likehoods), and trees were sampled every 100 generations. Bayesian posterior probabilities were calculated using a Markov chain Monte Carlo (MCMC) sampling approach implemented in MrBAYES 3_0b4 [28].

### Exposure of parasites to FQ pressure

Among the 33 field isolates tested for FQ susceptibility (see table 2 and [18]), two showed an IC_50 _higher than 100 nM (numbers 719 and 742) and one at 80 nM (number 747). Consequently, a concentration of 100 nM was selected for FQ pressure experiments, corresponding to the *in vitro *threshold adopted for resistance in field isolates studies [13,15,16]. The protocol, previously proposed by Cooper to obtain the CQ resistant *P. falciparum *strain 106/1, was used [21]. Before FQ pressure, parasites were grown to 5% parasitaemia at about 3% haematocrit in 60 ml of media. This parent culture was then split equally into 10 flasks in the first experiment and into 15 flasks in the second experiment, with fresh medium and red blood cells to bring the volume in each flask to 35 ml and about 3% haematocrit. Medium was replaced daily (or twice daily when parasitaemia was more than 5%). When parasitaemia had returned to about 7% to 15%, the culture medium was replaced by fresh medium containing 100 nM FQ (FQ medium). For the first week after drug application, cultures were monitored by Giemsa-stained thin blood films. Medium changes were performed daily under the same drug pressure conditions. After one week of FQ pressure, 50% of red blood cells were replaced, and fresh FQ medium was added. Then, FQ medium was replaced every three days and 50% of red cells were replaced every six days by fresh cells, for the duration of experiment (two months).

**Table 1:** Susceptibilities of four *P. falciparum *strains to CQ and FQ. IC_50 _and IC_90 _are given ± the standard deviation. The number of experiments is in brackets

		**IC_50 _(nM)**	**IC_90 _(nM)**
**Strain HB3**	**CQ**	21.83 ± 4.5 (10)	45.74 ± 13.9 (10)
	**FQ**	20.17 ± 6.0 (10)	28.61 ± 7.3 (10)
**Strain Dd2**	**CQ**	61.81 ± 28.7 (4)	166.75 ± 45.2 (4)
	**FQ**	18.88 ± 4.3 (4)	28.40 ± 7.9 (4)
**Strain 3D7**	**CQ**	12.6 ± 5.9 (33)	35.9 ± 16 (30)
	**FQ**	7.5 ± 3.3 (36)	13.1 ± 7.6 (36)
**Strain W2**	**CQ**	148.8 ± 60.3 (34)	> 500 (34)
	**FQ**	13.2 ± 4.1 (38)	29 ± 11.5 (35)

**Table 2:** Amino acid variations observed in PfCRT protein in 33 Cambodian isolates and laboratory strains within the phylogenic groups determined by Bayesian analysis. Composition of groups are as follows (laboratory clones and *field isolates *identified in [18]: group 1: *608*, *792*, 3D7, HB3; group 2: *738*; group 3: *602*, *613*, *634*, *702*, *734*, *736*, *770*; group 4: *722*, *742*; group 5: Dd2, W2, *536*, *572*, *631*, *637*, *643*, *647*, *654*, *665*, *666*, *671*, *683*, *685*, *691*, *693*, *716*, *717*, *719*, *739*, *747*, *749*, *794*.

	Residue numbers														
groups	74	75	76	144	148	194	220	271	326	333	356	371	N	IC_50 _CQ (nM)	IC_50 _FQ (nM)

Group 1	M	N	K	A	L	I	A	Q	N	T	I	R	4	19.88 (11.4–33.7)	26.59 (7.5–57.6)
Group 2	I	D	T	A	I	T	S	E	N	S	I	R	1	156.8	37.3
Group 3	I	D	T	F	I	T	S	E	N	S	I	R	7	95.51 (33.2–169.2)	26.34 (15.7–37)
Group 4	I	E	T	A	L	I	S	E	N	T	I	I	2	(91.8, 466.7)	(38.1, 120.2)
Group 5	I	E	T	A	L	I	S	E	S	T	T	I	22	149.17 (58.1–674)	39.13 (7.5–115)

In the first experiment, 0.86 × 10^10 ^parasites were exposed to 100 nM of FQ. A rapid decrease in the parasite population was observed and Giemsa smears were negative after four days. No parasites were detected throughout the two months of monitoring. In the second experiment, the number of parasites cultivated was about 4 fold higher than in the first experiment (2.76 × 10^10 ^parasites).

### Estimation of parasite populations by flow cytometry

To count the low parasite populations detected on Giemsa stained smears, an intraerythrocytic parasite double staining method using hydroethidine (HE) and thiazole orange (TO) was used as previously described [29]. Flow cytometric data acquisition and analysis were done on a FacsCalibur (Becton-Dickinson, San Jose, CA, USA). List mode data from 50,000 cells for control cultures and 10^6 ^cells for treated cultures were stored and processed with the CellQuest software.

## Results

### Responses to ferroquine of laboratory strains and field isolates and relation with PfCRT polymorphism or *pfcrt *gene expression

Four laboratory strains of *P. falciparum *(for which PfCRT sequences was known) were tested for their susceptibility to CQ and FQ. As expected (Table 1), CQ IC_50 _measurements showed that HB3 and 3D7 were CQ-sensitive, that Dd2 was moderately resistant to CQ, while W2 was the most CQ-resistant. In addition, all strains presented lower IC_50 _to FQ (7.5 nM to 20.17 nM) than to CQ except for HB3 which responded equally to the two drugs.

Concerning the complete set of 155 isolates tested in Cambodia for FQ susceptibility, five isolates (3.22%) had an IC_50 _> 70 nM and two (1.3%) had an IC_50 _> 100 nM. Comparison of IC_50 _of field isolates showed a significant correlation (r^2 ^= 0.3016) between CQ and FQ responses. Among this 155 isolates, data concerning *pfcrt *gene sequence and gene expression measurements done by Real Time PCR were available for 33 of them. In the subset selected for the present study, 51% of isolates were CQ sensitive (see legend of Table 2 of the present report and [18] for further details). The IC_50 _geometric mean (GM) observed were respectively 103.4 nM (range: 11.4 to 674 nM) for CQ and 31.5 nM (range: 7.50 to 120.2 nM) for FQ. Moreover, the five isolates displaying a FQ IC_50 _higher than 70 nM, and previously found in the total 155 isolates were present in this subset. This explains the higher correlation (r^2 ^= 0.58) between CQ and FQ responses observed for these 33 isolates. It was previously shown that there was no correlation between the K76T mutation and susceptibility to FQ in a group of Cambodian isolates [17]. On another hand, a relationship was demonstrated between the level of resistance to CQ and peculiar PfCRT haplotypes [18]. In order to examine whether all mutations occurred on PfCRT protein could be responsible of higher FQ IC_50_, A Bayesian phylogenic analysis was performed on available data [18]. The reconstructed phylogenic tree (data not shown) identified five groups numbered 1 to 5, each highly supported by a Bayesian posterior probability of 100%. Mutations in the PfCRT protein corresponding to these five groups are shown in Table 2 (group 1 represents the CQ sensitive laboratory strains or isolates and other groups the CQ resistant one). In this table, data concerning CQ and FQ IC_50 _are reported (calculation of geometric means for FQ IC_50 _were carried out for groups 1, 3 and 5). A large overlap observed between IC_50 _values, including the group 1, suggesting that none of PfCRT haplotypes analyzed could account for the variation in FQ sensitivity in the population studied. To study the possible role of PfCRT expression on FQ susceptibility, the relation between FQ IC_50 _and expression levels of *pfcrt *gene in field isolates using real time PCR (data not shown) was studied. The correlation observed (r^2 ^= 0.0011) was not significant indicating that the expression level of *pfcrt *mRNA was not correlated with the FQ IC_50_. These results are in line with those related to previous studies concerning expression level of *pfcrt *mRNA and CQ response [18].

### Experimental *in vitro *FQ pressure on W2 strain

**Figure 2:**
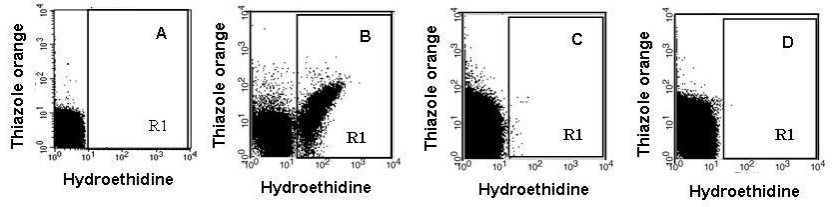
**Detection of parasites by flow cytometry analysis**. Parasites were double stained with hydroethidine and thiazole orange as described previously [28]. Fluorescence was expressed in Fluorescence Arbitrary Units (FAU) defined by the equipment. **A**: red blood cell profile. **B**: Untreated control culture. Red blood cells containing double stained (viable) parasites were visualized in R1. **C**: Parasites were cultivated without FQ pressure for 8 days. R1 represents the region where the parasite population is detected. Cells outside R1 (left) were unparasitized red blood cells. 38 parasites were counted in a total red blood cell population of 10^6^. **D**: Parasites were cultivated without FQ pressure for 36 days. Only 2 parasitized RBC were counted in R1 out of 10^6 ^red blood cells.

**Figure 3:**
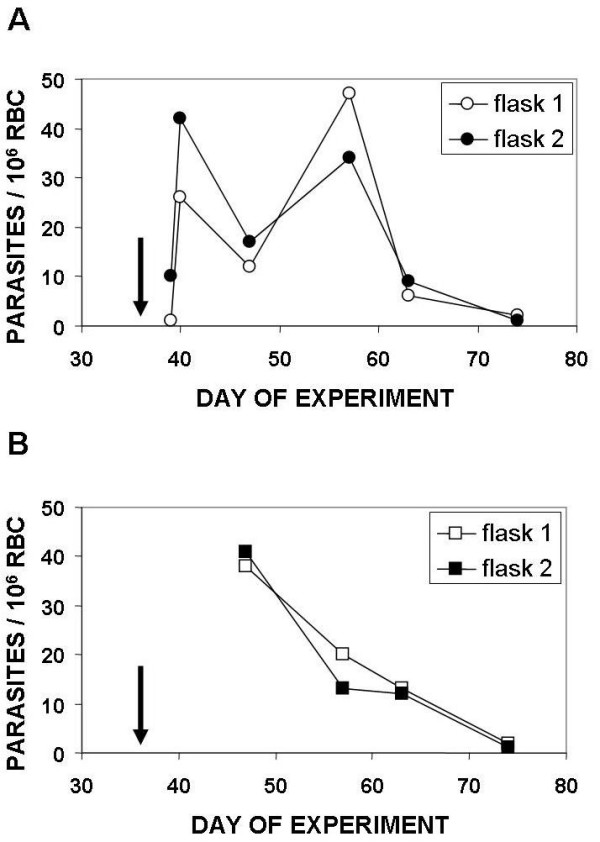
**Evolution of parasitaemia monitored by double staining flow cytometry in subcultures done from the two flasks in which surviving parasites were observed at day 36 in the main pressure experiment**. Arrows indicate the date in the main experiment at which surviving parasites were detected on Giemsa smears and confirmed by flow cytometry. A: Evolution in subcultures done under a continuous 100 nM FQ pressure. B: Evolution in subcultures done in the absence of FQ pressure.

Some IC_50 _values indicated a decreased response to FQ in rare field isolates ([17] and the present study). Consequently, induction of resistance in a *P. falciparum *laboratory strain under continuous 100 nM FQ pressure was tried using the experimental approach already used to obtain the CQ resistant clone 106/1 [21]. Previous studies had shown that *P. falciparum *clones exhibited wide variations in ability to acquire resistance to drugs [19]. The W2 strain acquired resistance to some drugs with 10 to 100 fold higher frequency than other clones [19]. In previous studies on African isolates [13,15,16], a resistance threshold of 100 nM was adopted for FQ. Among the 33 field isolates subset used in this study, some of them displayed FQ IC_50 _close or higher than 100 nM. This concentration was then selected for the pressure experiment. FQ pressure was initiated in two different experiments using the W2 strain. No parasites were observed throughout the two months of the first experiment, starting from 0.86 × 10^10 ^parasites. During the second experiment, starting from 2.76 × 10^10 ^parasites, parasites were undetectable based on Giemsa smears observations at day five due to the very low survival rate, but the follow up of cultures under 100 nM of FQ showed very few parasites on Giemsa smears at day 36. These parasites were viable because they converted hydroethidine into ethidium, which enabled a monitoring of their evolution in subcultures in the presence or absence of FQ, using double staining flow cytometry (Figure 2) [29]. In fact, this very low number of surviving parasites were unable to grow efficiently either in the presence or in the bsence of FQ and disappeared five weeks after the initiation of subcultures (Figure 3).

## Discussion

Ferroquine (SSR97193), currently in Phase I, is soon to be tested in patients with uncomplicated *P. falciparum *malaria. Therefore, investigations into potential resistance to this drug candidate are necessary. Among all studies already devoted to the effects of FQ on field isolates, apparent lower responses to this drug were observed mainly in areas of multidrug resistance [17]. The present study shows that the frequency of these decreased responses concerns about 3.22% of 155 Cambodian isolates if resistance threshold is fixed at 70 nM and about 1.3% if it is fixed at 100 nM. In the present study on *P. falciparum *field isolates or laboratory strains, it was possible to distinguish five polymorphic groups in PfCRT protein by a Bayesian phylogenic analysis. The examination of CQ and FQ IC_50 _in these five groups (Table 2) indicates that none of the different genotypes is directly associated with a decreased sensitivity to FQ. On the other hand, the level of expression of *pfcrt *mRNA is not correlated with the variations in FQ IC_50_. Insertion of the ferrocene moiety in the CQ lateral side chain results in modifications in the volume and shape of the molecule, as well as in its lipophilicity (logP = 5.1 versus 4.6 for CQ), its weak base properties and its electronic profile [10,11]. It is possible that these changes confer a low affinity of FQ (in comparison with CQ) for PfCRT which appears to be extremely structure-specific [30,31]. As a consequence, CQ resistant strains should be no more likely than others to develop FQ resistance. The drug pressure experiments done on W2 disproved this hypothesis. It has been impossible to recover a clone able to develop even in the absence of FQ in the culture medium. It seems that for surviving parasites, the cost of resistance to FQ may be too high in term of fitness [32-34]. In experimental conditions used, the frequency of parasites able to transiently survive to a sub-lethal dose of FQ is about 1 out of 10^10^. By comparison, experimental CQ pressure on the *P. falciparum *strain 106/1, which lacked only one mutation (K76T) on *Pfcrt *gene to acquire CQ resistance, led to a frequency of resistant parasites of about 10^-9 ^[21]. This mutation, which is due to a transversion of A to C and targeting the second base of the codon, is considered to be rare. Another study on the DHFR gene showed that the mutation rate of the gene at a given position occurred at less than 2.5 × 10^-9 ^mutation/DHFR gene/replication [35]. It was proposed that the resistance to CQ occurred four times in total over the whole history of this drug [36]. Because more than one mutation has been necessary to provide a viable resistant genotype, it can be expected that a hypothetical resistance to FQ would develop at a low frequency.

Another known candidate for mediating potential resistance to FQ might be the P-glycoprotein homolog 1 (Pgh1) protein encoded by the *pfmdr1 *gene. All studies pointed to an absence of correlation between FQ and mefloquine responses in field isolates from Cambodia [17], Senegal [16] or Gabon [15]. However, the question of *pfmdr *gene polymorphism or its copy number in isolates should be addressed to definitely rule out the possibility of a link between this marker and the response to FQ [37].

Analysis of the PlasmoDB database showed that other transporters were present in the parasite and might be involved in potential resistance to antimalarials [38-40]. It cannot be excluded that a resistance to FQ could develop in the future from a transporter encoded by one of these genes.

Recently, it has been suggested that the rapid development of drug resistance in the W2 strain could also be related to a defective DNA repair in this parasite [41]. Using this strain and despite this ability, it was not possible to select a viable FQ-resistant line. This may be due to the number of mutations which may be necessary to promote the binding and the transport of FQ by a putative transporter.

## Conclusion

The present results show that FQ susceptibility of 33 Cambodian *P. falciparum *field isolates was not related neither to phenotype of PfCRT protein nor to the level of gene expression. This indicates that CQ-resistant parasites as well as susceptible may be succumbing to FQ and confirms previous results observed on laboratory strains and field isolates. Pressure experiments done on W2 strain showed that the frequency of occurrence of FQ resistance is low in the experimental conditions used and that the cost of FQ resistance for the parasite is probably very expensive in term of fitness. This may be a limiting factor for spreading of potential FQ resistant parasites. In previous studies, it was shown that FQ security index, based on its cytotoxicity on L5178Y lymphoma cells, remained close to 700 whatever the CQ resistance level of *P. falciparum *used, when CQ security index falled from >1850 to >400 according to the CQ resistance of the strains [11]. FQ, given subcutaneaously or orally cured equally mice infected by CQ-sensitive or CQ-resistant *P. vinckei vinckei *strains at a dose of 8.4 mg·kg^-1^·day^-1 ^for four days with no adverse effects observed [11]. On the basis of these promising results, together with its capacity to kill *P. falciparum in vitro *and to protect against rodent malaria, FQ is likely to provide a valuable new treatment to bypass the CQ resistance of *P. falciparum*.

## Authors' contributions

WD and HJ were directly involved in the experimental work on parasites. TF provided the results on field isolates. BP established the sequence of W2 *pfcrt *gene. DD, LF, TF, JK and JB developed the experimental design. EV helped in logistics and performed the phylogenic analysis of PfCRT protein sequences, DD, WD, CB and JK wrote the manuscript. LF and JB obtained funding for this work. LP, TF, BP, LF, JB, provided critics and comments and proposed modifications prior manuscript submission.
